# A Mathematical Model for the Sounds Produced by Knuckle Cracking

**DOI:** 10.1038/s41598-018-22664-4

**Published:** 2018-03-29

**Authors:** V. Chandran Suja, A. I. Barakat

**Affiliations:** 1 Department of Chemical Engineering, Stanford University, CA-94305 California, USA; 2 Hydrodynamics Laboratory (LadHyX), CNRS UMR 7646, E´ cole Polytechnique, 91128 Palaiseau, France

## Abstract

The articular release of the metacarpophalangeal joint produces a typical cracking sound, resulting in what is commonly referred to as the cracking of knuckles. Despite over sixty years of research, the source of the knuckle cracking sound continues to be debated due to inconclusive experimental evidence as a result of limitations in the temporal resolution of non-invasive physiological imaging techniques. To support the available experimental data and shed light onto the source of the cracking sound, we have developed a mathematical model of the events leading to the generation of the sound. The model resolves the dynamics of a collapsing cavitation bubble in the synovial fluid inside a metacarpophalangeal joint during an articular release. The acoustic signature from the resulting bubble dynamics is shown to be consistent in both magnitude and dominant frequency with experimental measurements in the literature and with our own experiments, thus lending support for cavitation bubble collapse as the source of the cracking sound. Finally, the model also shows that only a partial collapse of the bubble is needed to replicate the experimentally observed acoustic spectra, thus allowing for bubbles to persist following the generation of sound as has been reported in recent experiments.

## Introduction

Some can and some cannot, but in any case knuckle cracking is very common. Knuckle cracking, the sound accompanying the articular release of the metacarpophalangeal (MCP) joint, has attracted the attention of researchers since the early 1900’s. Some of the earliest comprehensive studies on cracking of the MCP joint established that all joints cannot be cracked and that joints once cracked cannot be cracked again for approximately 20 minutes^1^. These observations have been confirmed by a number of studies over the years^2–5^; however, interestingly the source of the cracking sound during articular release continues to be debated^4^.

During articular release, the articulating surfaces (in this case the metacarpal and the proximal phalange) spring apart rapidly past the normal physiological range^6^. Roston and Wheeler hypothesized that this rapid motion of joints sets up vibrations in tissues leading to the cracking sound^1^. Mennel, on the other hand, ascribed the sound to the sudden tightening of the fibrous capsule about the joint during articular release^7^. However in 1971, Unsworth and co-workers through extensive experiments concluded cavitation and the subsequent collapse of cavitation bubbles in the synovial fluid as the source of the cracking sound^2^. Cavitation as the source of the cracking sound was widely accepted for over 40 years^3,6^, until Kawchuk and co-workers challenged this view recently by providing new evidence for the persistence of gas bubbles in the synovial fluid long after the cracking sounds were observed^4^. They hypothesized that tribonucleation-mediated sudden growth of bubbles and not their collapse was responsible for the sound.

The recent hypothesis of tribonucleation-mediated cavity inception as the source of the cracking sounds cannot explain the observed magnitude of the sounds^4^, suggesting the need for more detailed studies to resolve the origin of sounds during knuckle cracking. Experimental resolution to this problem is limited by the temporal resolution of available imaging techniques such as radiography (discrete)^1,2^, cineradiography (120 fps)^5^ and MRI (3.2 fps)^4^. Even today the capability of physiologically safe cineradiography (~100 fps)^8^ and MRI (80–100 fps)^9^ are far below 1200 fps, the minimum temporal resolution required to resolve the high speed dynamics of knuckle cracking as suggested by Watson *et al*.^5^, thus highlighting the need for numerical and theoretical approaches. The only study in this direction was conducted by Kanagawa and Taira, where they used the Rayleigh-Plesset equation to resolve the dynamics of cavitation bubbles subjected to impulsive and periodic forcing by an external pressure field^10^. Their results showed that cavitation bubbles under impulsive forcing can produce sharp sounds, highlighting the importance of bubble dynamics and underscoring the potential of numerical and theoretical approaches for understanding knuckle cracking.

In this manuscript, we develop a physiologically consistent mathematical model that can explain the generation of sounds accompanying knuckle cracking and thus aid in resolving the debate over the source of the sound. The model accounts for the dynamics of cavitation bubble collapse in the synovial fluid, as the presence of bubbles in the joint during knuckle cracking is unambiguously supported by virtually all researchers^1,2,4,5,10^ despite their differences on the origin of the sound. The cavitation bubble is assumed to originate due to the low pressure generated by tribonucleation and to subsequently evolve as the synovial fluid pressure changes inside a 2D approximated MCP joint. The acoustic waveforms and spectra generated by the collapsing bubble are shown to be consistent with our experimental recordings of knuckle cracking acoustics. Hence, this study establishes that the acoustic signature of cavitation bubble collapse is consistent with experimentally observed sounds, thus lending support for cavitation bubble collapse as a potential source of the sound. It also demonstrates the potential of detailed numerical and theoretical studies in cracking the enigma of the knuckle cracking sounds.

## Modelling

We model the sounds accompanying knuckle cracking by resolving the acoustic signature of cavitation bubbles inside the joint during articular release. For developing the model, the joint is assumed to be axisymmetric, thus simplifying the solution of the governing equations. The governing equations themselves describe three important phenomena: (1) the generation of transient low pressures during tribonucleation, (2) the dynamics of a newly formed cavitation bubble in the time-varying ambient pressure field, and (3) the simultaneous acoustic pressure field generated by the bubble.

### 2D Approximation of the MCP Joint

**Figure 1:**
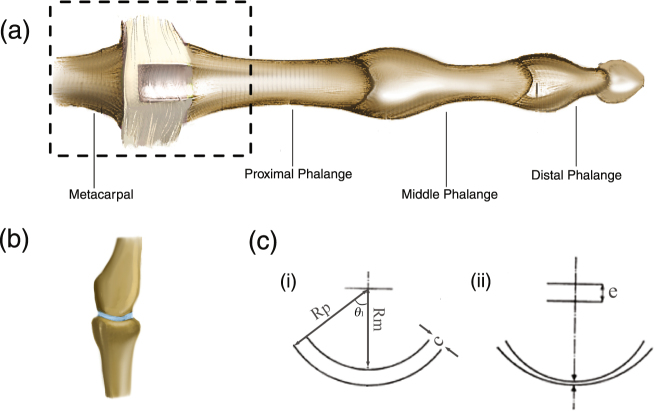
(**a**) The third metacarpophalangeal (MCP) joint^18^. (**b**) Rendered image of the MCP joint as seen through MRI^4^ (synovial fluid has been added for clarity). (**c**) 2D approximation of the MCP Joint^2^. Panel (i) shows the definition of the radii and the joint clearance. Panel (ii) shows a schematic of the 2D approximation of the MCP joint, where the centres of the two circular arcs are separated with an eccentricity *e*.

Geometric studies of the metacarpal head and the base of the proximal phalange of the MCP joint (Fig. 1a) have demonstrated that the geometry can be approximated by two spherical surfaces^2,11^. Exploiting the geometric symmetry, the joint can be approximated in two dimensions as two concentric circular arcs eccentrically positioned as depicted in Fig. 1c. The radii of the metacarpal head and the proximal phalange of the middle finger have been reported to be 6.44 ± 1.08 mm and 11.46 ± 2.30 mm, respectively^11^. Thus, in the simulations, we use an MCP joint geometry having a metacarpal radius (*R*_*m*_) of 6.44 mm and a proximal phalanx with a base radius (*R*_*p*_) of 11.46 mm. This gives the difference between the two radii *c* as 5.02 mm. Finally, the joint is assumed to be separated by 1.6 mm at the onset of cracking (before the joints have completed separated) as experimentally observed by Unsworth *et al*.^2^, which fixes the initial eccentricity *e*_0_ of the joint at 3.42 mm.

### Tribonucleation: Source of Cavitation Bubbles

Cavitation can initiate in the synovial fluid due to tribonucleation^12^ and has been hypothesized to be responsible for bubble formation during knuckle cracking^4^. The experimental observation that joints with an initial (at rest) spacing larger than approximately 1.4 *mm* cannot be cracked^2^ further suggests that cavitation in the MCP joint is indeed initiated due to tribonucleation.

The origin of tribonucleation-triggered cavitation is the viscous adhesion of a fluid between surfaces separated by a fluid. As the surfaces are forced apart, low pressures develop in the entrapped fluid. The closer the two surfaces are to one another prior to separation, the more effective is tribonucleation in triggering cavitation as lower pressures are generated. For an applied load *w*, the drop in pressure *p*(*θ*) at any angular location in a fluid between two spherical surfaces (as shown in Fig. 1c) can be expressed as^2^:

1 $$p=\frac{w}{2\pi {R}_{m}^{2}A}[\frac{1}{{\mathrm{(1}-\varepsilon cos(\theta ))}^{2}}-\frac{1}{{\mathrm{(1}-\varepsilon cos({\theta }_{1}))}^{2}}]$$

where,

2 $$A=\frac{1}{\varepsilon (1-\varepsilon )}+\frac{\mathrm{ln}(1-\varepsilon )}{{\varepsilon }^{2}}-\frac{cos({\theta }_{1})}{\varepsilon (1-\varepsilon cos({\theta }_{1}))}-\frac{\mathrm{ln}(1-\varepsilon cos({\theta }_{1}))}{{\varepsilon }^{2}}-\frac{sin{({\theta }_{1})}^{2}}{2{(1-\varepsilon cos({\theta }_{1}))}^{2}}$$

and

3 $$\varepsilon =\frac{e}{c}$$

where *θ*_1_ is the half angular sector of the joint and is taken as *π*/4 for computations. This model for pressure inherently assumes that the pressure equilibrates sufficiently rapidly in the synovial fluid so that the pressure in the synovial fluid is entirely governed by the load acting on the joint.

For a cracking joint, the eccentricity *e* becomes a function of time, thus making *ε* and hence *p* functions of time. We postulate that in going from rest to the final velocity observed in experiments^5^, the joint undergoes a constant acceleration *a* so that:

4 $$e(t)={e}_{0}-\frac{1}{2}a{t}^{2}$$

The acceleration *a* is determined from experimental data^5^ by assuming that between the two measurements of the joint spacing immediately before and after cracking, the joint would have accelerated to its final position. We hypothesize that the effects of acceleration are probably not reflected on the measured joint separation data^5^ due to the limited time resolution of the joint spacing measurements (8 *ms*) compared to the time scale of the entire phenomenon (10 *ms*).

We further observe that the lowest pressures are generated at the centre of the geometry. Thus, it is reasonable to assume that cavitation first occurs at the centre (*θ* = 0) and that the dynamics of the cavitation bubble are governed by the ambient pressure variation near this point. This further simplifies the analysis as it retains the axial symmetry of the problem enabling the use of the simplified form of the Rayleigh-Plesset equation for modelling the dynamics of the cavitation bubble. Hence, we use Eqs 1 (with θ = 0) and 4 to determine the ambient pressure variation inside a cracking joint.

### Bubble Dynamics

Once a cavitation bubble is formed, it responds to the ambient pressure variation. The dynamics of a spherical bubble of radius *R* in an infinite fluid domain is governed by the Rayleigh-Plesset equation obtained from the mass and momentum conservation equations^13,14^. For the purposes of modelling, we assume the bubble formed inside the joint to be spherical (and to remain spherical), and that its dynamics are not influenced by the surrounding geometry. We further assume that the vapour pressure varies very little in response to the temperature changes inside the bubble and hence the bubble is inertially controlled with the gas in the bubble having a constant polytropic index *k*. It is also known that the gases released during cavitation, 80% of which is CO_2_^2^, take time to be reabsorbed into the synovial fluid^2^ and hence mass transfer is neglected. Under these assumptions, the Rayleigh-Plesset equation simplifies to:

5 $$\frac{{P}_{v}-p(t)}{\rho }+\frac{{p}_{Go}}{\rho }{(\frac{{R}_{o}}{R})}^{3k}=R\frac{{d}^{2}R}{d{t}^{2}}+\frac{3}{2}{(\frac{dR}{dt})}^{2}+\frac{4\nu }{R}\frac{dR}{dt}+\frac{2S}{\rho R}$$

where *P*_*v*_ is the vapour pressure, *ρ* the density, *ν* the kinematic viscosity and *S* the surface tension coefficient of the synovial fluid. The solution of Eq. 5 yields the temporal variation of the bubble radius in response to the variation in ambient pressure. Considering the rapid changes in bubble volume, it is reasonable to assume adiabatic behaviour of gases inside the bubble and set the polytropic index *k* to *γ*, the ratio of specific heats of CO _2_ taken to be 1.3. The initial partial pressure *p*_*Go*_ inside the bubble is determined from the surface tension balance,

6 $${p}_{Go}=p\mathrm{(0)}-{P}_{v}+\frac{2S}{{R}_{0}}$$

To close the above equations, we need to specify the initial bubble radius *R*_0_. We choose a realistic initial bubble radius (200 *μm*) as is done in standard computations^14^.

### Acoustics

Even though the dynamics of the cavitation bubble are completely specified by Eqs 1 and 5, we need to reconstruct the acoustic pressure field to recover the sound generated. The oscillations of the bubbles produce acoustic pressure waves. The magnitude of these pressure waves at a distance *r* from the centre of the bubble whose volume *V*(*t*) is changing with time is given by the following equation^14^:

7 $${P}_{a}=\frac{{\rho }_{m}}{4\pi r}\frac{{d}^{2}V}{d{t}^{2}}$$

where *P*_*a*_ is the acoustic pressure and *ρ*_*m*_ is the density of the medium traversed by the acoustic waves. For standard values of the physical quantities (${\rho }_{m}=1015\,kg/{m}^{3},\,r=0.01\,m$), the sound pressure levels from our simulations are about 83 *dB*. This is comparable to the sound intensity measured experimentally by accelerometers attached to the skin^3^. We note, however, that the exact values of the constants *ρ*_*m*_ and *r* are inconsequential to our study as we non-dimensionalize the acoustic waves by their maximum amplitude in order to focus on the characteristic signature of the signal.

## Results and Discussion

### Ambient Pressure Variation

**Figure 2:**
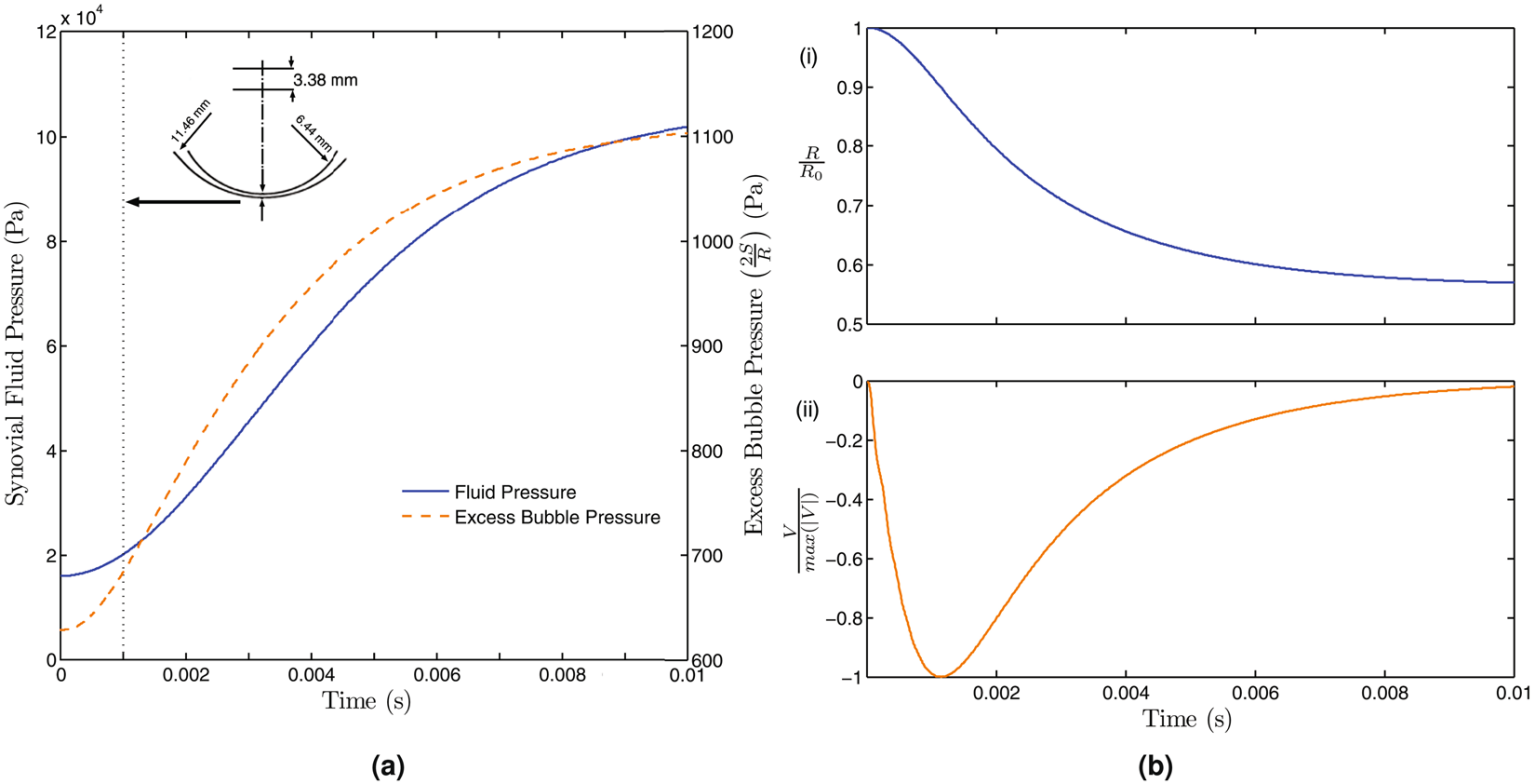
(**a**) Variation of the ambient pressure in the joint and the excess pressure of the bubble inside the joint from the solution of Eqs 1 and 5, respectively for an acceleration *a* of 72 *m*/*s*^2^. The inset shows the eccentricity of the joint at 1 *ms*, the time up to which an excellent match was obtained with experiments. (**b**) Solution of the Rayleigh-Plesset equation (Eq. 5) for the ambient pressure obtained from Eq. 1 for an acceleration *a* of 72 *m*/*s*^2^. (i) Evolution of the normalized bubble wall radius for the simulated parameters. (ii) Evolution of the normalized bubble wall velocity.

The ambient pressure variation is obtained from Eq. 1 for the geometrical parameters specified in the modelling section. For the geometry considered, at a load of 13 N the pressure in the synovial fluid goes well below its vapour pressure required to initiate cavitation and is taken as the load on the synovial fluid for the baseline simulation. The evolution of the ambient pressure in the joint for the baseline simulation is shown in Fig. 2a. Clearly, almost all the changes in the ambient pressure occur within the first 0.01 seconds, making the model for the ambient pressure consistent with the previous experimental observations of Unsworth *et al*.^2^. Furthermore, the ambient pressure grows increasingly fast initially, before gradually saturating at the zero load pressure when the joint separation becomes large. This has important consequences for the dynamics of the cavitation bubble.

### Dynamics of the Cavitation Bubble

For resolving the dynamics of the cavitation bubble, the Rayleigh-Plesset equation was coupled to the obtained ambient pressure variation and solved for a bubble with an initial radius of 200 *μm*. The variation of the bubble radius and bubble wall velocity with time are shown in Fig. 2b. As expected, in response to the ambient pressure variation, the bubble radius decreases, indicating bubble collapse. The negative values of bubble wall velocity also reflect the collapse, although it is evident that the rate of change of this velocity decreases and changes direction. This occurs when the pressure inside the collapsing bubble builds up faster than the rate at which the ambient pressure increases (Fig. 2a), causing the bubble collapse to decelerate. It is worth noting that only the collapsing phase of the bubble (i.e. negative wall velocities) is simulated and that the bubble persists following a partial collapse.

### Acoustics

The collapsing bubble produces acoustic pressure waves. The magnitude of these waves is obtained by solving the acoustic pressure equation (Eq. 7) for the collapsing bubble assuming the measurement point to be 0.01 meters from the centre of the bubble (approximate separation of the microphone from the MCP joint in the experiments). The acoustic waveform obtained from the experiments displays a characteristic dominant peak followed by a series of damped oscillations. A peak magnitude of 83 *dB* and a dominant frequency of 129 *Hz* are obtained for the acoustic pressure waves from the baseline simulation, which is comparable to the experimental reports in literature^3^.

### Comparison with Experiments

**Figure 3:**
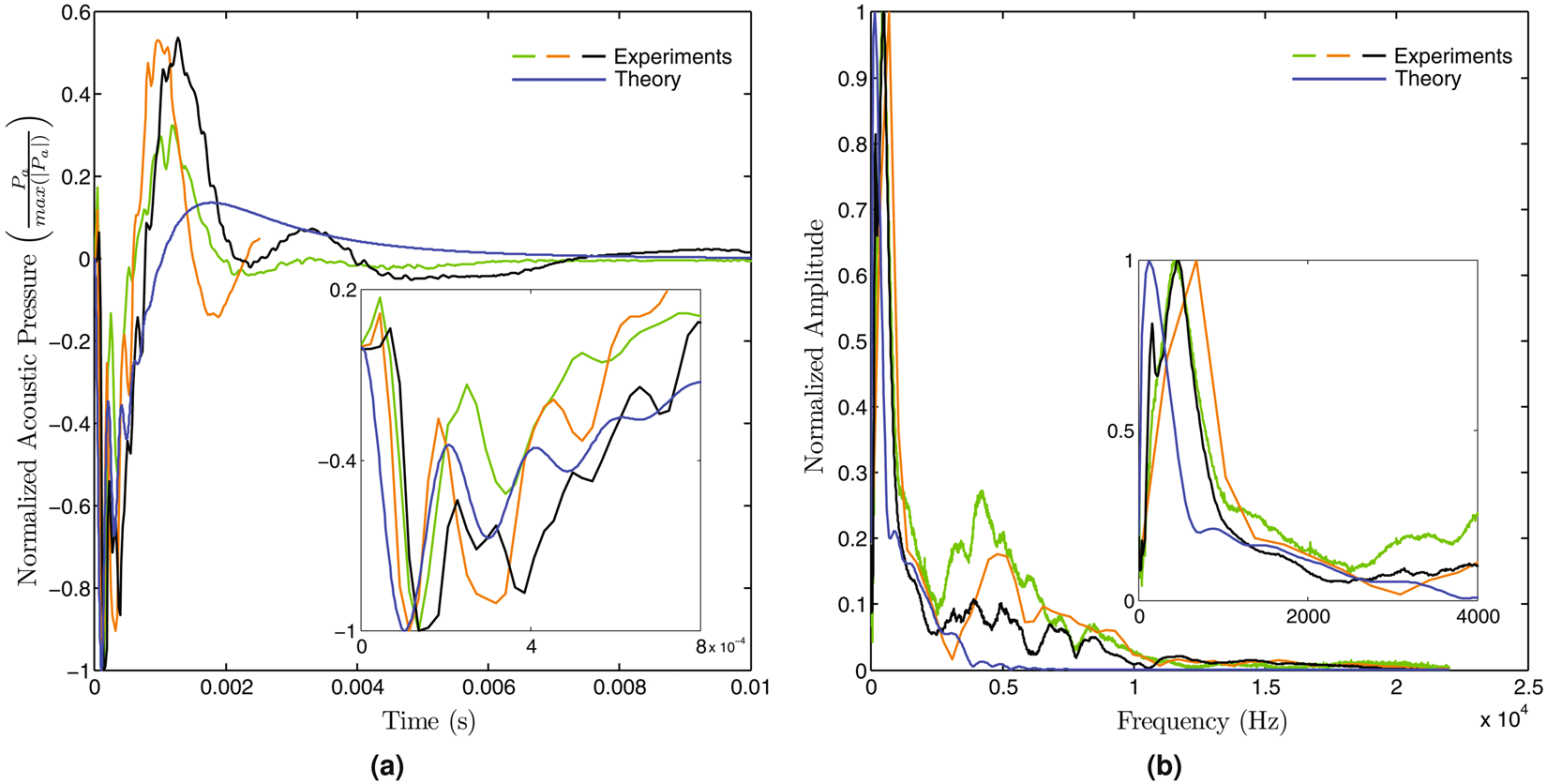
(**a**) Normalized acoustic pressure predicted by the model (blue curve) and measured experimentally (other curves). All curves exhibit an initial peak followed by a series of damped pressure oscillations. The inset illustrates the reasonably good agreement in the behaviour of the initial dominant peak which is responsible for the cracking sound. (**b**) Normalized amplitude-frequency spectrum for the simulation and the experiments. The inset shows that the dominant frequency obtained from the simulations is consistent with the frequencies obtained from the experiments.

Figure 3 depicts a comparison between the computed normalized acoustic pressure waveform and the waveforms obtained from experiments. The simulated results are seen to correlate well with the experimental measurements, exhibiting an initial dominant pressure peak around 0.1 *ms* (which as in Fig. 2a corresponds to an instantaneous eccentricity of 3.38 mm) after the initiation of the cracking followed by a series of damped pressure waves. The subsequent peaks after the initial dominant peak are however under predicted in the simulations. Nonetheless, the audio signature of the simulations compares very well with that of the experiments as the audio signature is dominated by the characteristics of the initial peak [See supplementary video]. Hence, given the variability in the acoustic pressure waveforms across subjects and even for the same subject at different instances (Fig. 3 green and black curves), the relatively simple model proposed here appears to be capable of capturing the essential features of the sounds accompanying knuckle cracking.

To further characterize these pressure waveforms, we performed a Fast Fourier Transform (FFT) and compared the frequency spectra of the simulations to those from experiments. Figure 3b demonstrates that the frequency at which the largest amplitude is observed is quite similar in the simulations and the experiments. Since the acoustic signature is dominated by the frequency (and its harmonics) having the highest amplitude, the acoustic signature of the simulations, as previously stated, is well correlated to those measured experimentally.

The good correlation between the simulations and experiments also suggests that bubble collapse is a plausible reason for the cracking sound. This result is in agreement with the conclusions of Unsworth *et al*.^2^. The competing theory of tribonucleation-mediated bubble inception as the source of the sound stems from the observation that stable bubbles remain in the synovial fluid after an audible sound is heard^4^. Interestingly, our results show that a partial collapse of the cavitation bubble leads to physiologically consistent acoustics and leaves behind a stable micro-bubble, suggesting that the cavitation theory of knuckle cracking acoustics could indeed explain the experimental observation of stable micro-bubbles. The final micro-bubble in our simulations remains unchanged infinitely long as we assumed an impervious bubble wall. However, future numerical studies may model bubble inception and employ a more realistic bubble wall permeability to confirm and study the inception, terminal and long term dynamics of these bubbles. If the the role of bubble inception on acoustics is tested and the presence of these terminal micro-bubbles after a cavitation bubble collapse is confirmed, all the experimental observations in the literature can be reconciled and thus the disagreements surrounding the source of the sound can be resolved.

### Sensitivity Analysis

To analyse the robustness of the model to the real-life variations of parameters across different knuckles, we performed a sensitivity analysis of the acoustic signal to the various parameters in the model including the joint acceleration, initial cavitation bubble radius, fluid viscosity, and joint spacing. To this end, we began by performing an order-of-magnitude analysis on the non-dimensionalized equations which revealed that the pressure term in the Rayleigh-Plesset equation had the strongest influence on the dynamics of the system (see Methods section). Hence, the parameters that influence the pressure term such as the joint acceleration, the load acting on the synovial fluid and the joint geometry are expected to have the largest effect on the acoustic signature.

**Figure 4:**
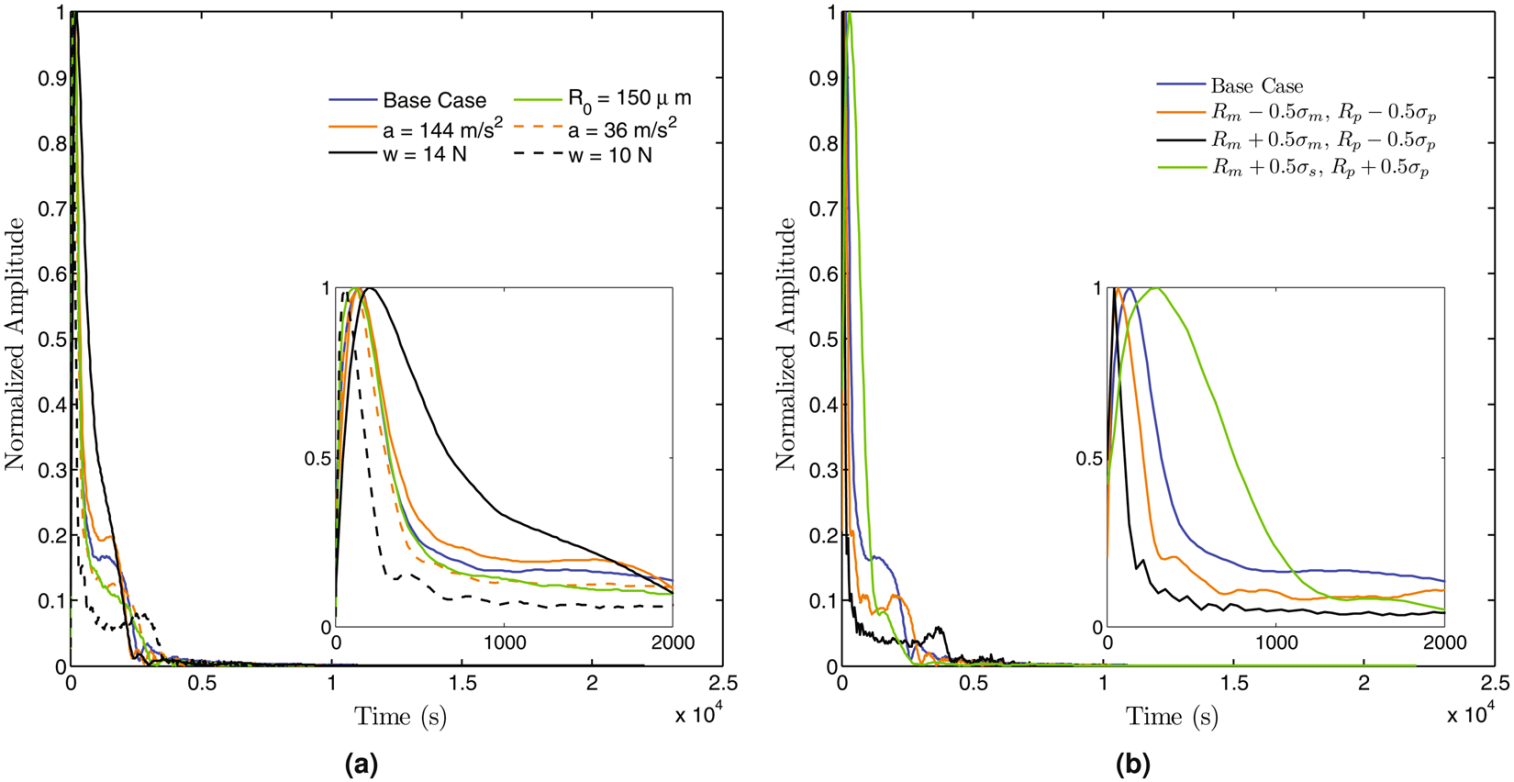
Sensitivity analysis of the model. (**a**) The model is sensitive to changes in the load but is relatively invariant to changes in acceleration and bubble radius. (**b**) The model gives physiologically consistent results but is also sensitive to changes in the geometry as shown by varying the geometry through half a standard deviation (*σ*_*m*_ = 1.08, *σ*_*p*_ = 2.30) above or below the mean metacarpal and proximal phalanx radii. The model diverged for the case of *R*_*m*_ − 0.5*σ*_*m*_, *R*_*p*_ + 0.5*σ*_*p*_ above a load of 6 *N*.

Figure 4a illustrates the dependence of the acoustic spectra on joint acceleration (*a*), the initial bubble radius (*R*_0_) and the load (*w*). The acceleration was varied from 36 *m*/*s*^2^ to 144 *m*/*s*^2^, accounting for the estimated experimental uncertainties in the spatial (±0.3 *mm*) and temporal (±0.002 *s*) resolution of the joint position^5^. An increase in the joint acceleration is seen to increase the relative magnitude of the high frequency components in the spectra, without affecting the dominant frequency. The frequency spectra are also relatively invariant to changes of ±100 *μm* in initial bubble radius. However, the acoustic signature is sensitive to changes in the applied load, with a change in the load from 10 *N* to 14 *N* changing the dominant frequency from 64 *Hz* to 193 *Hz*. More interestingly, the parametric study on the effect of the load showed that for every geometry considered, there was a limiting value of the load beyond which the model failed to converge. This limiting value is a function of the geometry (both the radius of curvatures and the half angular extent of the joint) and for the cases we considered, we found the maximum limiting value to be around 15 *N* or about 15% of the experimentally measured external load at which the joint cracks^1,2^. Acoustic signatures close to the experiments were obtained for a load of ~13 *N*, which is slightly less than this maximum limiting value. We hypothesize that this threshold provides a ballpark estimate for the hypothesis advanced by Unsworth *et al*.^2^ that only a fraction of the applied external load acts on the synovial fluid, with the rest being taken up by the surrounding tissue.

Figure 4b shows the parametric sweep of the joint geometry assuming constant joint separation (the initial joint eccentricity varies between 0.52 and 0.72). For a joint having the metacarpal and proximal phalanx one half standard deviation either above or below the mean^11^, our model yielded results comparable to the experiments, except for one case (*R*_*m*_ − *σ*_*m*_, *R*_*p*_ + *σ*_*p*_). For this physiologically rare case (the radius of the articulating surfaces usually change proportionately), the model fails above an applied load of 6 *N*. Finally for completeness, a sweep of the other parameters including synovial fluid viscosity (which is known to change with age and disease) showed that the dynamics were relatively insensitive to these parameters as suggested by the dimensional analysis.

It is reassuring that the relatively simple model presented here yields results that closely match experimental findings, especially in the initial phase of knuckle cracking, thus underscoring the potential of numerical simulations in understanding knuckle cracking. Relaxing some of the assumptions in the model are expected to yield even better results. The model makes a number of assumptions including a 2D approximation of the joint, approximations to the pressure field, a simple acceleration profile for joint separation, an axisymmetric cavitation bubble, negligible effects of confinement on the bubble dynamics, the presence of only a single bubble, and that the acoustics are solely due to bubble collapse. One or more these assumptions, especially that of the presence of a single cavitation bubble, could very well explain why the model predictions do not closely match experiments after the dominant acoustic pressure peak. Multiple cavitation bubbles may occur in reality, but the first bubble will be expected to originate close to the centre where the lowest pressures are located. Logically, the initial dynamics will be governed by the primary bubble before interactions from other collapsing bubbles begin to influence the sound. Moreover, as the pressure drops in the centre of the joint, there will be a flow of synovial fluid from the surroundings to this low pressure region, altering the pressure field. This effect may initially be negligible but may have a significant influence on the dynamics at a later stage. The initial minor positive peak seen in the acoustic pressure measured from experiments (Fig. 3) could be from the growth phase of the bubble or from other sources^6^, none of which were accounted for in our model.

## Conclusions

We have developed a mathematical model accounting for the dynamics of collapsing cavitation bubbles in a cracking metacarpophalangeal joint for identifying the source of sounds accompanying knuckle cracking. The magnitude and the dominant frequency of the sounds predicted by this model are in close agreement with measurements reported in literature. Furthermore, the acoustic waveforms predicted by the model are shown to be in close agreement with our experiments, especially in the vicinity of the dominant peak.

The success of this relatively simple mathematical model fulfils two goals relevant to the current debate surrounding the origin of the sounds accompanying knuckle cracking. Firstly, the good correlation between the simulations and the experiments establishes support for cavitation bubble collapse as a potential source of the cracking sound. Secondly, the success of the model underscores the potential of detailed numerical simulations in resolving the origin of the sounds. Future numerical simulations may be aimed at accurately simulating the inception, terminal and long term behaviour of cavitation bubbles. If such studies test the acoustic signature of bubble inception and confirm the existence of stable micro-bubbles after a cavitation bubble collapse, they would contribute significantly towards resolving the ongoing debate about the origin of knuckle cracking sounds.

## Methods

### Experiments

The acoustic signature of knuckle cracking from three subjects in their early twenties were recorded at Ecole Polytechnique’s Acoustics Laboratory. The sounds were recorded in an anechoic chamber using a AV-JEFE TCM 160 microphone which was connected to a laptop computer running Audacity sampling the input data at 44 kHz. The microphones were positioned less than 0.01 meters away from the MCP joint. All experiments were carried out in accordance with relevant guidelines and regulations approved by the IRB. Informed consent was obtained from all the subjects.

### Physiological Considerations

To develop a physiologically relevant model for the sounds accompanying knuckle cracking, it is important to understand the anatomy of the MCP joint and the properties of the synovial fluid in the joint. The MCP joint is the joint between the metacarpal bones and the phalanges of the fingers, more commonly referred to as the knuckles. As seen in Fig. 1a, the metacarpal head fits into the base of the proximal phalange, and the two structures are held together by the transverse metacarpal ligaments. For the purpose of modelling, the ligaments and tendons are neglected and only the metacarpal head and the base of the proximal phalange are considered for the joint geometry (Fig. 1a).

The synovial fluid is a viscous and non-Newtonian fluid that occupies the space inside the joint. For the purpose of modelling, the physical properties of the synovial fluid reported in literature were used. The synovial fluid is known to be a shear thinning fluid with a dynamic viscosity (*μ*) of 0.4–0.5 *Pa*-*s* at low shear rates and ∼0.1*Pa*-*s* at high shear rates^15^. The density (*ρ*) of the synovial fluid is in the range of 1008–1015 *kg*/*m*^3^ ^12^, while the surface tension coefficient (*S*) has been reported to be 46.7 ± 5.7 *mN*/*m*^16^. The vapour pressure (*P*_*v*_) of the synovial fluid is 6500 *Pa*^12^.

### Numerical Solutions

Eqs 1 and 5 were solved to obtain the variation of the volume of the cavitation bubble with time. Subsequently, Eq. 7 was used to recover the acoustic pressure signature of the bubble. All the equations were solved using MATLAB ^®^ (R2010b, The MathWorks, Inc., Natick, Massachusetts, United States).

As Eq. 5 is a stiff ordinary differential equation, for improved accuracy, a non dimensional form of the equation (See Appendix Eq. 8) is solved using the ODE15s solver in MATLAB with an error tolerance (absolute and relative) of 10^−7^ for a duration of 0.01 s, the typical duration of the life of a cavitation bubble inside the joint^2^. Using a simple backward difference scheme, we obtain the acceleration of the bubble wall, which we use to compute the second temporal derivative of volume. Subsequently, using Eq. 7, we obtain the acoustic field.

For generating actual sounds from the computed pressure pulses, we re-sampled the data in MATLAB to record the data consistently into a wav file. MATLAB scripts were also written to process the data obtained from the experiments. All audio files were then processed in Audacity to play back the sound (See supplementary video).

#### Validation and Accuracy of Numerical Solution

The Rayleigh-Plesset equation is a non linear second order ordinary differential equation that can become stiff depending on the initial conditions and the variation of the pressure in the domain. Our codes were validated to ensure the physical and numerical accuracy of the solutions.

**Figure 5:**
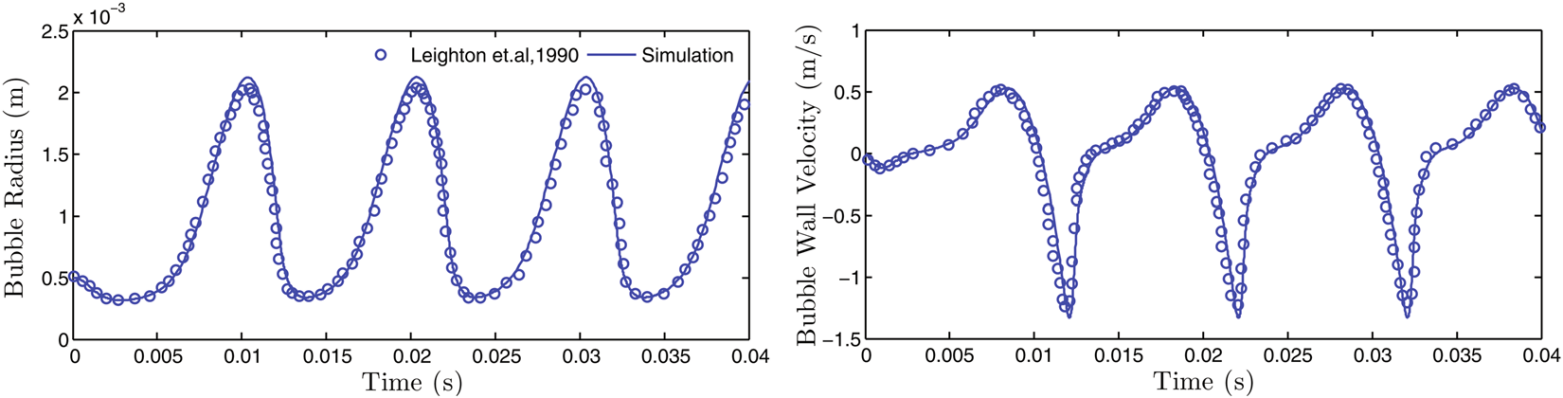
Validation of the solution procedure against data from Leighton *et al*.^17^.

The physical accuracy of the solution procedure was evaluated by simulating a test case for which experiments were conducted by Leighton *et al*.^17^. The simulated solutions are in very good agreement with the results in literature (Fig. 5), confirming the accuracy of the solution procedure.

The numerical accuracy of the solutions presented in this paper were evaluated by computing the repeatability of the numerical solutions across different ODE solution routines in MATLAB. The base case was solved using two different stiff ODE solvers, ODE15s and ODE23s, with different error tolerances until the solutions across the solvers were identical up to the floating point relative accuracy in MATLAB.

#### Non-Dimensional and Order-of-Magnitude Analysis

We non-dimensionalize the governing equations by choosing the average separation of the joint prior to cracking as the reference scaling for length (*l*), while the average duration of a typical knuckle crack is chosen as the reference scaling for time (*τ*). To obtain a convenient scale for pressure, the external load at which a knuckle cracks is chosen as the scaling for force (*w*_0_), yielding $\frac{{w}_{0}}{{l}^{2}}$ as the scaling for pressure. Using the above scaling, we obtain the following non-dimensional formulation of the governing equations:

8 $$\begin{array}{c}\frac{{d}^{2}\tilde{R}}{d{\tilde{t}}^{2}}=\frac{{w}_{0}{\tau }^{2}}{\rho {l}^{4}}[\frac{{\tilde{p}}_{v}-\tilde{p}(\tilde{t})}{\tilde{R}}+\frac{{\tilde{p}}_{Go}}{\tilde{R}}{(\frac{{\tilde{R}}_{o}}{\tilde{R}})}^{3k}]+\frac{3}{2}[\frac{1}{\tilde{R}}{(\frac{d\tilde{R}}{d\tilde{t}})}^{2}]\\ \quad \quad +\,\frac{4\nu \tau }{{l}^{2}}[\frac{1}{{\tilde{R}}^{2}}\frac{d\tilde{R}}{d\tilde{t}}]+\frac{2S{\tau }^{2}}{\rho {l}^{3}}[\frac{1}{{\tilde{R}}^{2}}]\end{array}$$

where,

9 $$\tilde{p}(\tilde{t})=\frac{\tilde{w}}{2\pi {\tilde{R}}_{m}^{2}A}[\frac{1}{{\mathrm{(1}-\varepsilon cos(\theta ))}^{2}}-\frac{1}{{\mathrm{(1}-\varepsilon cos({\theta }_{1}))}^{2}}]$$

10 $$A=\frac{1}{\varepsilon (1-\varepsilon )}+\frac{\mathrm{ln}(1-\varepsilon )}{{\varepsilon }^{2}}-\frac{cos({\theta }_{1})}{\varepsilon (1-\varepsilon cos({\theta }_{1}))}-\frac{\mathrm{ln}(1-\varepsilon cos({\theta }_{1}))}{{\varepsilon }^{2}}-\frac{sin{({\theta }_{1})}^{2}}{2{(1-\varepsilon cos({\theta }_{1}))}^{2}}$$

11 $$\varepsilon =\frac{\tilde{e}}{\tilde{c}}\quad {\rm{and}}\quad \tilde{e}={\tilde{e}}_{0}-\frac{1}{2}\tilde{a}{\tilde{t}}^{2}$$

For the typical values of the scales ($l\sim 0.001\,m$, $\tau \sim 0.01\,s$, ${w}_{0}\sim 100\,N$), the order of magnitude of the coefficients of the different terms in Eq. 8 are as follows:

$$\frac{{w}_{0}{\tau }^{2}}{\rho {l}^{4}}\sim {\mathscr{O}}\mathrm{(4)}$$

$$\frac{4\nu \tau }{{l}^{2}} \sim {\mathscr{O}}\mathrm{(1)}$$

$$\frac{2S{\tau }^{2}}{\rho {l}^{3}} \sim {\mathscr{O}}\mathrm{(1)}$$

Clearly the quantities that modify the pressure term, which include the load and the acceleration of the joint, have the most significant impact on the dynamics of the system as predicted by our model (Fig. 4).

### Data availability

All data generated or analysed during this study are included in this published article (and its supplementary video).

## Electronic supplementary material

Supplementary Video
